# Enhanced pneumothorax visualization in ICU patients using portable chest radiography

**DOI:** 10.1371/journal.pone.0209770

**Published:** 2018-12-21

**Authors:** Julia Ley-Zaporozhan, Hassan Shoushtari, Ravi Menezes, Leon Zelovitzky, Devang Odedra, Laura Jimenez-Juan, Karyn Brunet, Yasser Karimzad, Narinder S. Paul

**Affiliations:** 1 Department of Medical Imaging, University Health Network, Toronto General Hospital, Canada; 2 Department of Radiology, University Hospital Munich, Ludwig-Maximilians University, Germany; 3 Department of Medical Imaging, Sunnybrook Health Sciences Centre, Canada; 4 Department of Medical Imaging, London Health Sciences Centre and St Josephs’ Healthcare, Robarts and Lawson Research Institutes, Canada; Homerton University Hospital, UNITED KINGDOM

## Abstract

**Objective:**

Pneumothorax development can cause precipitous deterioration in ICU patients, therefore quick and accurate detection is vital. Portable chest radiography is commonly performed to exclude pneumothoraces but is hampered by supine patient position and overlying internal and external material. Also, the initial evaluation of the chest radiograph may be performed by a relatively inexperienced physician. Therefore, a tool that could significantly improve pneumothorax detection on portable radiography would be helpful in patient care. The aim of this study was to evaluate the clinical utility of novel enhancement software for pneumothorax detection in readers with varied clinical experience of detecting/excluding pneumothoraces on portable chest radiographs in ICU patients.

**Subjects and methods:**

206 portable ICU chest radiographs, 103 with pneumothoraces, were processed with and without enhancement software and reviewed by 5 readers who varied in reading experience. Images were grouped for different complexity levels.

**Results:**

The mean AUC for pneumothorax detection increased for 4/5 readers from 0.846–0.957 to 0.88–0.971 with a largest improvement for the reader with least experience. No significant change was noted for the reader with the longest reading experience. The image complexity had no impact on the interpretation result.

**Conclusion:**

Pneumothorax detection improves with novel enhancement software; the largest improvement is seen in less experienced readers.

## Introduction

More than 5.7 million patients are admitted to an Intensive Care Unit (ICU) in the United States every year [1–3]. The principal cause of ICU admission is cardiorespiratory insufficiency and these patients have a guarded prognosis due to multiple co-morbidities, limited respiratory reserve, multiple organ failure and sepsis. Patients may become acutely obtunded due to respiratory complications that include lung atelectasis, lung consolidation and development of a pneumothorax. The treatment for each of these conditions is very different, therefore accurate diagnosis is essential. Although it is important to diagnose each of these conditions, the development of a pneumothorax can result in rapid patient deterioration, especially if the patient is intubated and ventilated [4].

Portable chest radiography is the most common imaging modality in ICU patients [5], however it has significant technical challenges for accurate demonstration of a pneumothorax due to limitations such as superimposition of anatomical structures, sub-optimal patient positioning, lung hypo-inflation, limited patient cooperation, and obscuration of anatomical features due to chest tubes, cardiac monitoring equipment and vascular lines. As ICU patients are often unstable, it is difficult to transport them away from the ICU environment and therefore it is important to evaluate strategies for improving the detection of a pneumothorax using portable chest radiography. In addition, the initial evaluation of a portable chest radiograph may be performed by a relatively inexperienced reader, and this may cause additional complexity in the accurate and timely detection of a pneumothorax [6, 7].

The purpose of this study was to evaluate the clinical performance of novel enhancement post processing software in the demonstration and detection of pneumothoraces on portable digital chest radiographs performed in ICU patients.

## Materials and methods

### Patients

The study was approved by the institutional ethics committee (Research Ethics Board of the University Health Network, Toronto, Canada; REB number: 10-0871-AE). The individual patient consent was not obtained because the data were analyzed anonymously.

A prospective trial in which all portable ICU (cardiovascular and general ICU) chest radiographs performed at a single tertiary center over a 3 months period were collected, resulting in 1427 consecutive studies.

The chest radiographs were collected on a dedicated personal computer; and only the first conventional portable chest image (index chest radiograph) per patient was taken for review purposes. All of the patients from which index chest radiographs were selected had a longitudinal analysis of existing portable chest radiographs (and any chest CT scans) performed subsequent to the index chest image and during the patients’ ICU admission, to confirm the presence or absence of a pneumothorax on the index radiograph.

Two subspecialty trained chest radiologists, with more than 18 years’ experience and 7 years’ experience of reading chest radiographs respectively, analyzed the original images for the presence or absence of pneumothorax first separately and then in consensus. Only the cases with agreement were selected for the study. Only two unequivocal cases occurred during the preselection process.

Age and gender distribution were not considered to be an issue as the selected patients were used as their own controls.

### Complexity score

The size of the pneumothorax was characterized into small (<2 cm interpleural distance), medium (2–4 cm) and large (>4 cm interpleural distance) by the two radiologists in consensus. The selected images (with and without a pneumothorax) were scored for image complexity. This score used a linear scale to rate image quality, patient size, body rotation, presence of tubes and lines (like intercostal tubes, oxygen supply tubing, vascular, ECG and pacemakers’ line) due to superimposition onto the lungs. Each metric was scored (0–3) to indicate increased image complexity (Table 1). The accumulative score for each radiograph was 0 (slim patient, no rotation, no tubes or lines and excellent image quality) to 12 (obese patient, severe rotation, > 4 tubes or lines, poor image quality).

**Table 1:** Each metric was scored between 0–3, 0 = best, 3 = worst. The accumulative metrics of image quality were combined to form an image complexity score.

Complexity Score				
Score	0	1	2	3
**Patient Size**	Slim	Average	Large	Obese
**Patient Rotation**	None	Slight	Moderate	Severe
**Tubes/Lines/Catheters**	0	1	2–4	>4
**Image Quality**	Excellent	Good	Average	Poor

From the original cohort of 1427 studies 206 ICU chest radiographs were selected, 103 radiographs containing a pneumothorax and 103 without pneumothorax, with a representative spectrum of image complexity usually found in ICU patients (Table 2). The radiologists who performed the image selection and categorization did not participate further in image analysis.

**Table 2:** Each radiograph was characterized by the presence of pneumothorax, the size of pneumothorax and image complexity.

	Image characteristics				
Complexity Score	Pneumothorax				No Pneumothorax
	*Small*	*Medium*	*Large*	Total	
**0**	1	2	1	4	2
**1**	7	9	8	24	7
**2**	10	5	8	23	16
**3**	8	4	2	14	16
**4**	6	3	5	14	11
**5**	6	6	0	12	14
**6**	2	3	2	7	20
**7**	1	1	1	3	14
**8**	0	1	0	1	2
**9**	0	0	0	0	1
**10**	0	1	0	1	0
**11**	0	0	0	0	0
**12**	0	0	0	0	0

### Image post processing

The enhanced chest radiographs were created using commercially available Carestream Pneumothorax Visualization Software (Carestream Health, Rochester, NY) [8]. This software combines multi-frequency band processing with a contrast-limited adaptive histogram equalization algorithm that operates over small regions in the image, rather than the entire image. This approach enables enhancement of subtle details throughout the entire image with the added benefit of local contrast enhancement. The local contrast is adaptively adjusted such that the contrast in non-homogeneous regions is enhanced while the contrast in more homogeneous regions is limited. This emphasizes the textural differences between the characteristic markings for the regions inside and outside of the lung; accentuating the appearance of free air in the chest cavity [6, 9].

Each chest radiograph was post processed using the edge enhancement software to create an enhanced (E-CXR) image. The post processing was done automatically without user interaction. Therefore, for each patient there was a conventional portable digital chest radiograph (C-CXR) and an enhanced (E-CXR) image. All radiographs were anonymized, assigned a study ID and loaded onto a dedicated research workstation.

### Image interpretation

All radiographs were read on a DICOM GSDF calibrated 3mP diagnostic quality display (RadiForce G33, Monochrome LCD Monitor) in a dedicated reading room.

Five readers (R1-R5) with variable experience in interpreting chest radiographs were selected: R1 (chest radiologist with 30 years’ experience); R2 (chest radiologist with 3 years’ experience); R3 (radiology research fellow); R4 (technologist with more than 13 years’ experience of performing portable radiography); and R5 (non-medical research fellow in our imaging department with no experience in reading chest radiographs, but who received basic training in detecting pneumothoraces).

A calibration exercise was undertaken with each reader using 10 portable chest radiograph pairs (C-CXR and E-CXR), these images and scores did not form part of the final evaluation. Each reader independently reviewed anonymized chest radiographs presented in random order and indicated their level of confidence in detecting a pneumothorax for each lung separately using a 5-point scale: 1 = high likelihood of no pneumothorax; 2 = likely no pneumothorax; 3 = uncertain; 4 = likely there is a pneumothorax; and 5 = high likelihood of a pneumothorax. A score of 1, 2 or 3 was taken to indicate the absence of a pneumothorax, a score of 4 or 5 was taken to indicate the presence of a pneumothorax.

**Fig 1:**
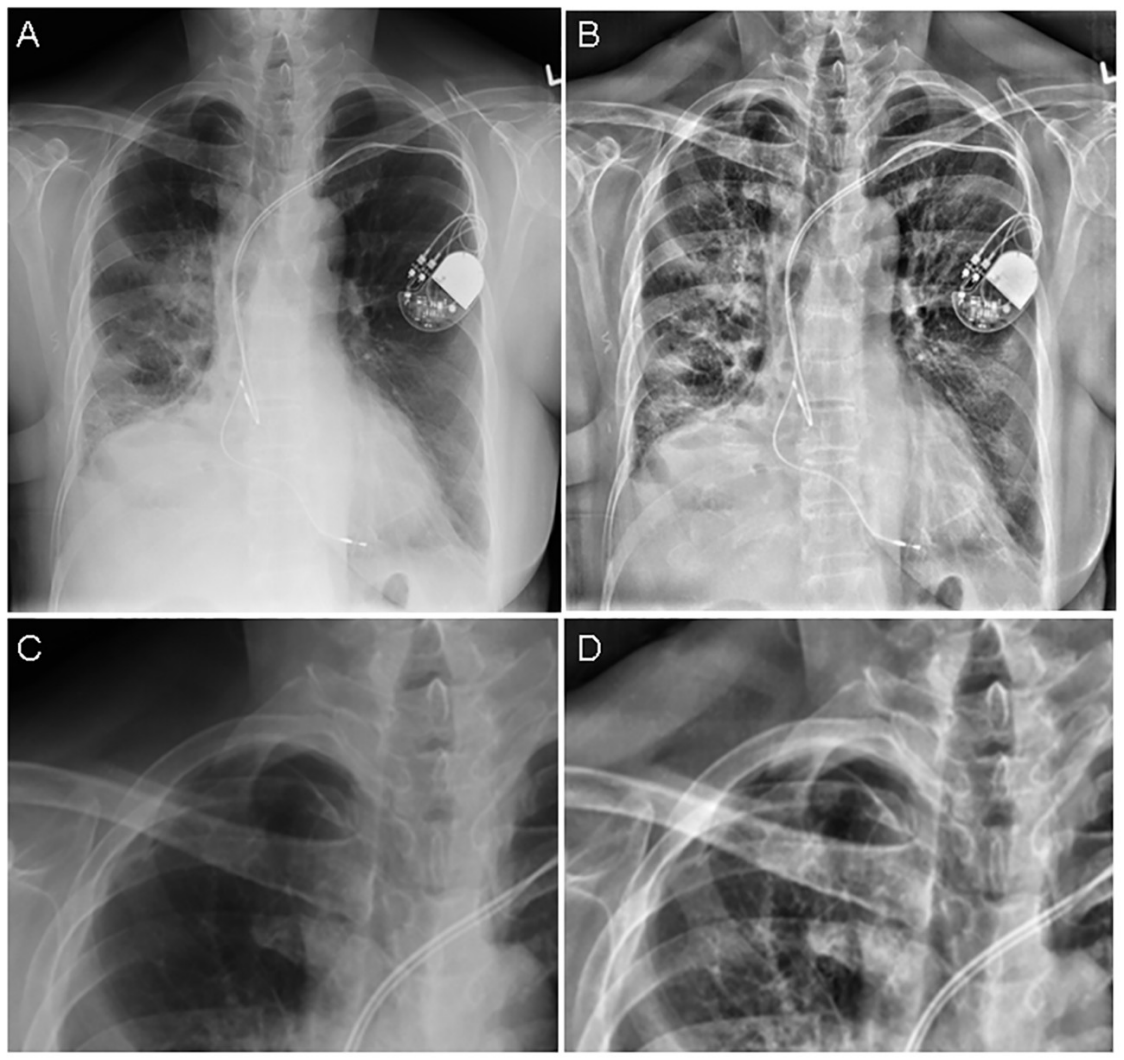
Portable chest radiograph of intensive care unit patient demonstrating the appearances of the conventional (A) and edge enhanced (B) images with corresponding magnified views demonstrating a small right apical pneumothorax (C, D).

First, each reader independently scored each radiograph (both lungs) for the presence/absence of pneumothorax on the C-CXR only (read 1). After a washout period of 4 weeks per reader a re-evaluation of the C-CXR followed (read 2). In read 2 after the C-CXR had been scored for a second time, the E-CXR was made available alongside the corresponding C-CXR and the combination was scored for the likelihood of a pneumothorax (read 3) using the same 5-point grading scale. As the presentation of the C-CXR and the E-CXR are very obviously different (Fig 1), it was not possible to blind the readers to the post processing software when used. After read 3 each reader was asked whether they preferred the conventional image only, edge enhanced image only, or both images for the specific task of detecting a pneumothorax on a case by case basis.

The results from read 1 were used to assess inter-observer variation; read 2 results determined intra-observer variation and read 3 results indicated the added value of the E-CXR above that of the C-CXR for each reader in detecting a pneumothorax. Therefore, all 5 readers evaluated a total of 206 radiographs with 412 lungs in each of three reading sessions that provided a total of 5 x 412 x 3 = 6180 lungs for analysis.

The time taken for reading each image and making a decision on the presence or absence of a pneumothorax was also recorded in each instance. The reading time was assessed to test for any increase in detection speed that might provide potential benefits for the patient due to faster detection and treatment of a pneumothorax. All data are given in S1 Table.

### Statistics

The sample size has been detected with a significance threshold of p < 0.05 (alpha-error) and a statistical power of 80% (i.e., beta error 20% or less) according to Hulley et al. [10]. The expected accuracy was set at the level of 90% and the assumed difference by using the enhanced software of greater than 5%. The calculated sample size was 407 lungs, resulting in 203.5 patients.

The readers entered their results into a form based on a Microsoft Access database (US). These data were transferred into a spreadsheet for statistical analysis using MedCalc Statistical Software (Version 17.9.7, MedCalc Software bvba, Ostend, Belgium. Sensitivity, specificity, positive and negative predictive values were calculated. Furthermore, the area-under-the-curve (AUC) was calculated and a comparison between the reads was performed using the Hanley & McNeil method [11, 12].

To assess the difference in reading time, a paired t-test was used (level of significance < 0.05).

## Results

### Inter-observer variation

The sensitivity, specificity, negative and positive predictive ratios for pneumothorax detection for all five readers during the first read of conventional chest radiographs is demonstrated in Table 3. Analysis of the receiver operator characteristics (ROC) for each reader revealed a range of performance as measured by the area under the curve (AUC) with a low value of 0.827 for reader 5 to the highest value of 0.971 for reader 3.

**Table 3:** Reader performance in pneumothorax detection using conventional chest radiography and edge enhancement software reader performance in pneumothorax detection using conventional chest radiography (first and second read) and additional edge enhancement software (third read).

Reader	Sensitivity	Specificity	PPV	NPV	AUC (95% CI)
**First Read**					
**R1**	95.15%	89.32%	74.81%	98.22%	0.953 0.928 to 0.971
**R2**	83.50%	93.85%	81.90%	94.46%	0.931 0.902 to 0.953
**R3**	92.23%	95.79%	87.96%	97.37%	0.959 0.935 to 0.976
**R4**	93.20%	94.50%	84.96%	97.66%	0.961 0.938 to 0.978
**R5**	71.84%	91.59%	74.00%	90.71%	0.865 0.828 to 0.896
**Second Read**					
**R1**	79.61%	94.50%	82.83%	93.29%	0.923 0.893 to 0.947
**R2**	90.29%	95.79%	87.74%	96.73%	0.966 0.943 to 0.981
**R3**	90.29%	95.79%	87.74%	96.73%	0.955 0.930 to 0.973
**R4**	83.50%	97.09%	90.53%	94.64%	0.95 0.924 to 0.969
**R5**	64.08%	96.44%	85.71	88.96%	0.827 0.787 to 0.862
**Third Read**					
**R1**	92.23%	89.00%	73.64%	97.17%	0.934 0.905 to 0.956
**R2**	91.26%	95.47%	87.04%	97.04%	0.955 0.930 to 0.973
**R3**	94.17%	89.97%	75.78%	97.89%	0.971 0.950 to 0.985
**R4**	90.29%	94.82%	85.32%	96.70%	0.959 0.935 to 0.976
**R5**	71.84%	97.41%	90.24%	91.21%	0.88 0.845 to 0.910

### Intra-observer variation

Analysis of the second read demonstrated a general reduction in sensitivity but increase in specificity for detection of a pneumothorax with conventional chest radiographs (Table 3). I.e. for reader 4 the sensitivity dropped from 93% to 83% while the specificity increased from 94% to 97%.

### Influence of enhancement algorithm

In the third read, the E-CXR was viewed alongside the C-CXR. The enhanced image improved the sensitivity for pneumothorax detection for every reader compared to the second read. An increase in AUC values was found for readers 2–5, while reader 1 (longest reading experience) remained quite stable (Table 4). The largest improvement in performance was seen for reader 5 (least reading experience) with a difference of 3.4%. The number of the uncertain answers (score 3) decreased dramatically by using the additional E-CXR (Table 5).

**Table 4:** Comparison of reader performance for pneumothorax detection using conventional (first and second read) and additional edge enhanced radiographs (third read). P-value has been calculated between second read AUC and third read AUC using the Hanley & McNeil method.

Reader	First Read AUC	Second Read AUC	Mean AUC	Third Read AUC	Change in AUC	p-value
**R1**	0.953	0.923	0.938	0.934	-0.40%	0.5870
**R2**	0.931	0.966	0.9485	0.955	0.65%	0.4217
**R3**	0.959	0.955	0.957	0.971	1.40%	0.2377
**R4**	0.961	0.95	0.9555	0.959	0.35%	0.5106
**R5**	0.865	0.827	0.846	0.88	3.40%	*0*.*0270*

**Table 5:** The number of the uncertain answers (score 3) using conventional chest radiography (first and second read) and additional edge enhancement software (third read).

Uncertain Answers			
Reader	First Read	Second Read	Third Read
**R1**	100	143	17
**R2**	30	22	6
**R3**	19	39	18
**R4**	58	81	33
**R5**	20	55	10

### Time taken for image interpretation

For the first read, the readers needed (mean) 51 to 89 seconds, with the most experienced performing fastest. Readers R1 –R4 spent significantly less time (21–46%) performing the second read of the conventional chest radiographs, reader R5 spent 10% less time but with no significant difference (Table 6). The additional time taken to perform the read of the enhanced images ranged (mean) from 26 to 49 seconds. The combined time to perform the second and third reads was slightly longer than the time taken to perform the first read only.

**Table 6:** Time taken to perform reads (time in seconds; SD = standard deviation). p-value: t-test (paired samples). ■ = Read 1 compared to read 2. ▲ = Read 1 compared to time (read 2 + read 3).

Reader	First Read mean (SD)	Second Read mean (SD)	Mean difference	p-value ■	Third Read mean (SD)	p-value ▲
**R1**	51.1 (43.0)	28.3 (21.2)	- 22.8 (- 45%)	*<0*.*0001*	26.0 (13.8)	0.32
**R2**	61.6 (47.3)	48.4 (64.9)	- 13.2 (- 21%)	*0*.*017*	28.4 (42.3)	*0*.*02*
**R3**	88.6 (65.8)	47.9 (26.0)	- 40.7 (- 46%)	*<0*.*0001*	48.7 (47.2)	0.181
**R4**	63.0 (29.6)	43.9 (23.8)	- 19.1 (- 30%)	*<0*.*0001*	32.8 (22.2)	*<0*.*0001*
**R5**	69.8 (93.7)	62.5 (130.1)	- 7.3 (- 10%)	0.533	26.4 (55.4)	0.126

### Influence of image complexity on pneumothorax detection

A ROC analysis was performed that examined the association between Complexity Score and accuracy at the patient level. None of the AUC values were significantly different from 0.5, as indicated by their 95% CI. So, it does not appear as though complexity ‘predicts’ accuracy.

### Reader preference for image interpretation

**Fig 2:**
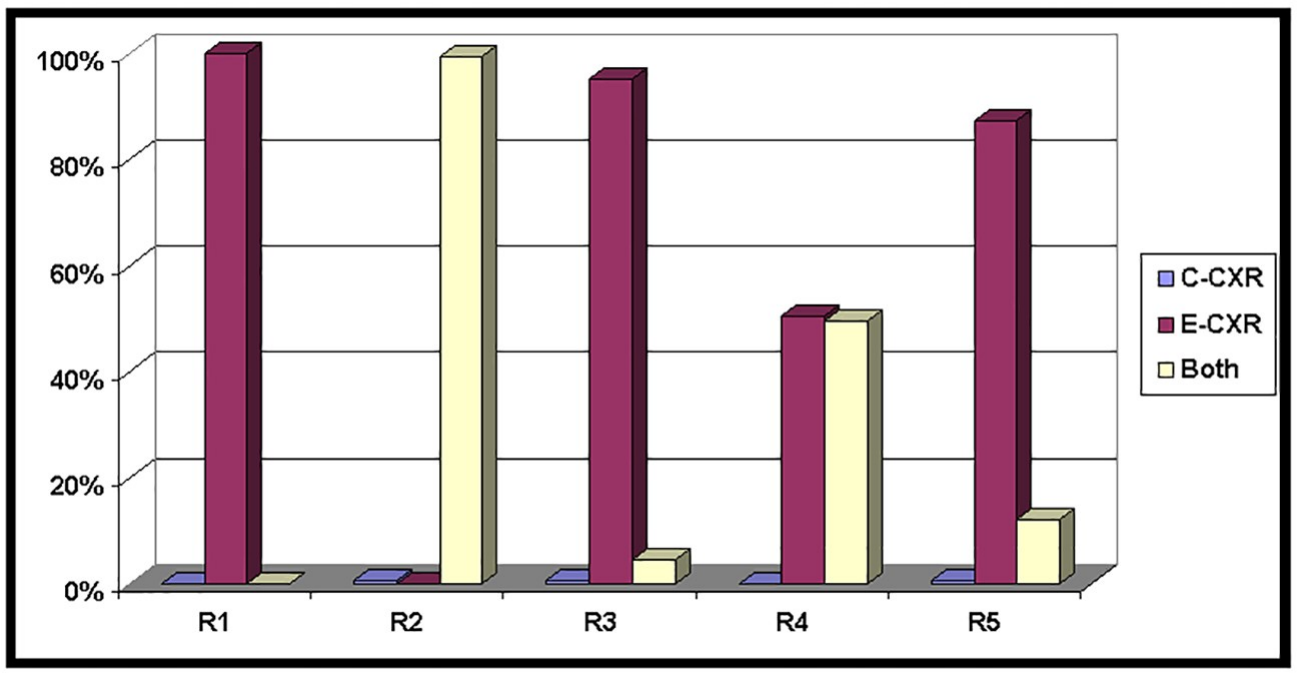
Reader preference for pneumothorax detection using conventional (C-CXR) or edge enhanced radiographs (E-CXR) or both.

All readers expressed a strong preference for the edge enhanced chest radiograph either in isolation or in combination with the conventional radiograph for pneumothorax detection (Fig 2).

## Discussion

Pneumothorax detection is essential in the ICU patient and optimization of portable chest radiographs is critical for this purpose.

We have described the implementation of enhancement software on portable ICU chest radiographs for assessment of pneumothoraces. The original conventional chest radiograph is unchanged (and displayed side-by-side) and the enhanced series acts as a companion image purely to assist in pneumothorax detection.

Using the enhanced CXR image, 4 out of 5 readers showed increased area under the curve for detection of a pneumothorax. The largest increase in accuracy was demonstrated by the least experienced reader from 0.846 to 0.88 (change 3.4%).

Interestingly the complexity of an image showed no impact on the detection rate of pneumothorax. However, it was noted, that the overall image quality was quite good, with most of the examinations achieving a score of 5 or less (88% of cases) and only 12% of examinations having a complexity score of 6 and above.

After an ICU film is taken, a quick analysis of major complications is required. In the academic setting, the initial read may be performed by a relatively junior member of the health care team and the formal report performed by an experienced chest radiologist may be available after a short delay. Therefore, there might be a delay in appropriate patient management if the pneumothorax is not detected or the patient may have unnecessary treatment if a pneumothorax is diagnosed in error. The results of our study suggest that the software has most utility in the hands of less experienced readers. Although this study did not engage readers from the ICU, it is likely that the observed trend of disproportionate benefit would be replicated for the ICU team.

To the best of our knowledge, there are no published studies that evaluate a software algorithm for pneumothorax enhancement. So far, the software has only been used for detection of tubes and lines [6].

Presently, there are many options to enhance image information in plain film images [13]. In this review the authors show that advances in electronics and computer technology have resulted in rapid development of digital image receptors and displays. The rapid development of image-processing techniques and advanced applications such as dual-energy and temporal subtraction radiography; digital tomosynthesis, computer-aided detection and diagnosis promise to substantially improve the future practice of chest radiography. This is especially true as the body habitus of patients becomes more challenging due to obesity. Uppot et al [14] demonstrated a 50% increase in examinations of limited x-ray quality over a 14 year period due to increase in body habitus. To overcome these limitations a substantial amount of research has been dedicated to the development of new detectors and imaging processing technologies. Hardware development necessitates significant investment in upgrading the fleet of installed portable radiography units whereas the image processing route is more versatile one as it can be implemented on an existing PACS system.

Different approaches for image post processing have been evaluated. Kheddache et al used different enhancement algorithms to increase image contrast, edge enhancement and linearization of the monitor contrast [15]. It was found, that aggressive image enhancement resulted in significant increases in image noise that obscured the enhancement effect and fine linear structures, such as a pneumothorax, could not be enhanced properly. This drawback was overcome by the software used in our study, as all readers rated the image quality of the enhanced CXR image superior to the conventional CXR image. However, it has to be kept in mind, that this evaluation was focused on detection of pneumothorax and not on the detection of other important pathologies such as lung consolidation.

The trial design allowed for a spectrum of image complexity; patient body habitus, patient positioning, a variable number of lines, tube and catheters and also varying overall image quality. We have also evaluated the utility of the enhancement software using a range of reader experience. The enhancement software did notsignificantly effect the sensitivity and specificity for pneumothorax detection for the most experienced reader, but the number of uncertain cases was reduced significantly using the enhancement software. Improved observer performance for pneumothorax detection was found in the other four readers with a more significant improvement—a change in ROC metrics of 3.4%—in the least experienced reader. This is an important finding as often the least experienced member of the medical or radiological team is the first to review portable chest radiographs and subsequent patient management is often determined by this interpretation. This was also the outcome experienced by a study that used automatic enhancement software for visualization of tubes and catheters on CXR images [16]. The use of an automatic imaging-processing algorithm reduced localization variability and enabled the medical interns to perform at approximately the same level as the chest radiologists.

However, in all readers the uncertain cases were reduced significantly by the use of the software. Here, especially the most experienced reader showed the largest impact of the software to reduce the number of unclear cases. Therefore, it can be said, that the software helps to increase the precision of the radiological report.

One limitation of the study is that that no ICU physician was included as a reader. The initial trial design was to enroll readers from the ICU, including the radiographer. However, despite initial enthusiasm, it was not possible to recruit the ICU readers during the period of this trial. Therefore, we adjusted the trial design to utilize readers with a wide range of experience in reading ICU chest radiographs for the presence of a pneumothorax to act as surrogate for the ICU team.

A second potential limitation of this study was the use of a conventional CXR as the reference standard. It would have been optimal to have all ICU patients examined by CT to get the most exact visualization of any thoracic abnormality. However, this was not feasible in our setting and this also does not reflect daily clinical practice where clinical decisions are made based on conventional CXR findings.

## Conclusion

Enhancement software improves pneumothorax detection in ICU patients when used to provide a companion image alongside a conventional portable chest radiograph and the improvement is most noticeable in less experienced observers. This software should be considered for routine use in all ICU patients, especially those patients suspected of having a pneumothorax.

## Supporting information

S1 Table This excel file summarizes all raw data information for each image regarding quality and rating. (XLSX)
